# Chemical Composition and Antioxidant Activity of *Euterpe oleracea* Roots and Leaflets

**DOI:** 10.3390/ijms18010061

**Published:** 2016-12-29

**Authors:** Christel Brunschwig, Louis-Jérôme Leba, Mona Saout, Karine Martial, Didier Bereau, Jean-Charles Robinson

**Affiliations:** UMR QUALITROP, Université de Guyane, Campus Universitaire de Troubiran, P.O. Box 792, 97337 Cayenne Cedex, French Guiana, France; christel.brunschwig@gmail.com (C.B.); louis-jerome.leba@univ-guyane.fr (L.-J.L.); mona.saout@gmail.com (M.S.); karine.martial@univ-guyane.fr (K.M.); didier.bereau@univ-guyane.fr (D.B.)

**Keywords:** *Euterpe oleracea*, açaí, antioxidant activity

## Abstract

*Euterpe oleracea* (açaí) is a palm tree well known for the high antioxidant activity of its berries used as dietary supplements. Little is known about the biological activity and the composition of its vegetative organs. The objective of this study was to investigate the antioxidant activity of root and leaflet extracts of *Euterpe oleracea* (*E*. *oleracea*) and characterize their phytochemicals. *E*. *oleracea* roots and leaflets extracts were screened in different chemical antioxidant assays (DPPH—2,2-diphenyl-1-picrylhydrazyl, FRAP—ferric feducing antioxidant power, and ORAC—oxygen radical absorbance capacity), in a DNA nicking assay and in a cellular antioxidant activity assay. Their polyphenolic profiles were determined by UV and LC-MS/MS. *E*. *oleracea* leaflets had higher antioxidant activity than *E*. *oleracea* berries, and leaflets of *Oenocarpus bacaba* and *Oenocarpus bataua*, as well as similar antioxidant activity to green tea. *E. oleracea* leaflet extracts were more complex than root extracts, with fourteen compounds, including caffeoylquinic acids and *C*-glycosyl derivatives of apigenin and luteolin. In the roots, six caffeoylquinic and caffeoylshikimic acids were identified. Qualitative compositions of *E. oleracea*, *Oenocarpus bacaba* and *Oenocarpus bataua* leaflets were quite similar, whereas the quantitative compositions were quite different. These results provide new prospects for the valorization of roots and leaflets of *E. oleracea* in the pharmaceutical, food or cosmetic industry, as they are currently by-products of the açaí industry.

## 1. Introduction

*Euterpe oleracea* (*E*. *oleracea*), known as açaí worldwide and wassaye in French Guiana, is a palm tree native from the Amazonian rainforest, which gives purple berries with high potential currently used as dietary food supplements or in cosmetics thanks to their high antioxidant activity. Traditionally, açaí berries are consumed as a beverage called “açaí vino” in Brazil. Since the mid 2000s, many scientific studies focused on *E*. *oleracea* berries due to its biological activities including anti-proliferative, anti-inflammatory, antioxidant, and cardioprotective properties. *E*. *oleracea* was also found to be extremely rich in antioxidant compounds including phenolic acids, flavonoids and anthocyanins [1,2].

While açaí berries are mainly used as foodstuffs, *E*. *oleracea* vegetative organs (such as leaves and roots) are traditionally used in medicine, curing snake bites [3], diabetes, kidney and liver pains, fever, anemia, as well as arthritis, or used as hemostatic [4,5]. Roots of *Euterpe precatoria*, a close species to *E*. *oleracea*, also showed anti-inflammatory [6] and antioxidant properties [7]. The vegetative organs of *E*. *oleracea* are considered as waste from the açaí palm heart industry and could be potential sources of phytochemicals with cosmetic, pharmaceutical or nutritional applications. Currently, there is interest in valorizing polyphenols-containing food wastes such as grape skins, olive mill waste water, citrus peels, seed wastes or berry leaves [8,9] as there is a growing demand for polyphenols for pharmaceutical, cosmetic and food industries, especially to treat cardiovascular, and skin diseases, cancer, aging, and diet issues. Some studies have already been carried out on açaí seeds, another waste from the açaí industry. Antioxidant activity [10] and anti-nociceptive activity have been demonstrated [11], showing that this waste could find applications in the food or pharmaceutical industries. Therefore, there is also a real value in enhancing the knowledge of the phytochemical composition and pharmacological activities of açaí roots and leaves. A previous study showed that roots and leaflets of two other Amazonian palm trees (*Oenocarpus bacaba* and *Oenocarpus bataua*) had high antioxidant activity, underlining both *Oenocarpus* vegetative organs as good sources of antioxidant compounds [12]. In this paper, we therefore investigated the antioxidant activity of root and leaflet crude extracts of *E*. *oleracea* using different chemical antioxidant assays (DPPH—2,2-diphenyl-1-picrylhydrazyl, FRAP—ferric reducing antioxidant power, and ORAC—oxygen radical absorbance capacity), a DNA nicking assay and a cellular antioxidant activity assay, while LC-MS/MS was performed to elucidate the structures of the bioactive compounds.

## 2. Results and Discussion

### 2.1. Antioxidant Activity

#### 2.1.1. Antioxidant Activity in the Chemical Assays (DPPH, FRAP, and ORAC)

Antioxidant properties of root and leaflet extracts from *E*. *oleracea* were investigated using different chemical assays (DPPH, FRAP and ORAC) having different modes of action: hydrogen transfer (ORAC), electron transfer (FRAP) or a mixed mode (DPPH) [13]. In all antioxidant tests, best results were obtained for acetone extracts independently of the organ used. Leaflet extracts were more active than root extracts, independently of the solvent used.

In the DPPH assay, values for *E*. *oleracea* leaflet extracts ranged from 480 to 990 µmol Trolox Eq/g Dry Matter (µmol TEq/g DM). DPPH activity of *E*. *oleracea* acetone leaflet extracts were at least two-fold higher than *E*. *oleracea* and *O*. *bataua* berries [14], two-fold higher than *Oenocarpus* leaflets acetone extracts [12], and slightly lower than green tea (1200 µmol TEq/g DM) (Table 1).

In the FRAP assay, all values for leaflet extracts ranged from 1000 to 1400 µmol Fe(II)Eq/g DM. They were two-fold higher than palm berries [14,15,16], at least two-fold higher than antioxidant foodstuffs like lettuce [17], but lower than green tea extracts (1900–2700 µmol Fe(II)Eq/g DM) (Table 1). When comparing leaflet acetone extracts, *E*. *oleracea* FRAP activity was two-fold higher than *O*. *bataua* and similar to *O*. *bacaba* [12].

The ORAC assay, considered as one of the most relevant amongst antioxidant chemical assays, gave ORAC values from 1600 to 2200 µmol TEq/g DM, for leaflet extracts. They were at least three-fold more active than palm berries in the ORAC assay [14], at least 1.5-fold more active than the best *Oenocarpus* leaflet extracts [12] and almost equivalent to green tea with 2400 µmol TEq/g DM (Table 1). Besides, even if *E*. *oleracea* roots were less active than *E*. *oleracea* leaflets in all the antioxidant tests, their activities were as high or higher than those of *E*. *oleracea* and *O*. *bataua* berries, as illustrated by ORAC results (300–1300 µmol TEq/g DM) (Table 1).

Root and leaflet extracts from *E*. *oleracea* were active across the board in the different chemical antioxidant assays (DPPH, FRAP, and ORAC) showing that they act according to different mechanisms of action such as electron and/or hydrogen transfer. Root and leaflet extracts were at least as active as palm berries, considered as super fruits. Leaflet extracts were the most antioxidant extracts, almost equivalent to green tea leaves, which have one of the strongest recorded antioxidant activity.

#### 2.1.2. Antioxidant Activity in the DNA Nicking Assay

**Figure 1:**
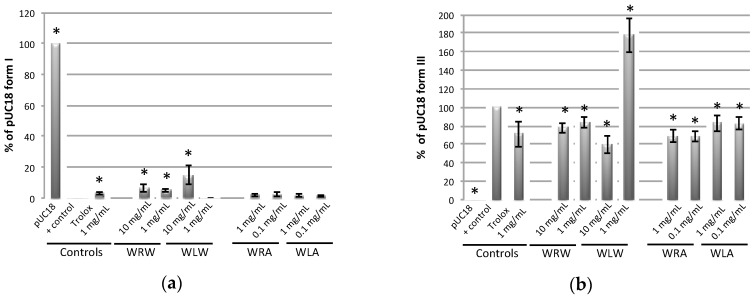
Antioxidant activity of *Euterpe oleracea* extracts in the DNA nicking assay indicating: (**a**) protection of supercoiled form I; and (**b**) formation of nicked linear form III; W: Wassaye (*E*. *oleracea*); R: roots; L: leaflets; W: water; A: acetone/water 70/30; (* *p* < 0.05; *n* = 3).

Despite optimization of the DNA nicking assay conditions, the sensitivity using methanol extracts was low [16], therefore only aqueous and acetone extracts of *E*. *oleracea* were assessed in the DNA nicking assay. All root extracts of *E*. *oleracea* were found to be active in this assay. The aqueous and acetone root extracts had an antioxidant effect at all concentrations tested; they protected form I from degradation and reduced the formation of linear nicked form III in a similar way to Trolox at 1 mg/mL (Figure 1). Aqueous leaflet extracts had an antioxidant effect in the DNA nicking assay at all concentrations tested (1 mg/mL and 10 mg/mL), while acetone leaflet extracts had an antioxidant effect at 10 mg/mL and a prooxidant effect at a lower concentration of 1 mg/mL. The overall high potency of the root and leaflet extracts of *E*. *oleracea* in the DNA nicking assay encouraged the evaluation of their antioxidant activity in an assay physiologically more relevant, i.e., a cell-based antioxidant assay.

#### 2.1.3. Cytotoxicity and Cellular Antioxidant Activity (CAA)

As root and leaflet extracts of *E*. *oleracea* were not cytotoxic to normal human dermal fibroblasts (NHDF) at 100 µg/mL in the MTT (3-(4,5-dimethylthiazol-2-yl)-2,5-diphenyltetrazolium bromide) assay (Table S1), they were tested at this concentration in the Cellular Antioxidant Activity (CAA) assay. 

Root and leaflet extracts from *E*. *oleracea* were quite active in the cellular antioxidant assay with the median effective concentration (EC_50_) ranging from 10 to 30 µg/mL (Table 1). Most of the palm extracts were more active than fruits including kiwis, blueberries, carrots and strawberries, which had a high antioxidant activity (EC_50_ from 50 to 200 µg/mL) in a similar cell-based assay [18].

Leaflet extracts of *E*. *oleracea* were more active in the CAA assay (45–208 µmol Quercetin Eq/g DM) than root extracts (10–30 µmol Quercetin Eq/g DM) and acetone extracts were more active than methanol extracts. Activities for *E*. *oleracea* were quite similar to *Oenocarpus* roots (10–50 µmol Quercetin Eq/g DM), whereas *E*. *oleracea* leaflets were at least two-fold superior than *Oenocarpus* leaflets (7–100 µmol Quercetin Eq/g DM) [12]. Interestingly, there was a very high correlation between the ORA activity and the cell-based activity (Pearson’s r correlation coefficients of 0.976) (Figure S1), which are both based on the ROO· radical scavenging capacity of the extracts. These correlations were also found in the work of Girard-Lalancette et al., 2009 and Leba et al., 2016 [12,18]. The high potency of extracts in the ORAC assay translated well in the CAA assay, meaning that compounds were able to act in a cellular environment, either by crossing membrane cells or by directly scavenging radicals outside the cells. These results encourage investigating the chemical composition of *E*. *oleracea* root and leaflet extracts.

### 2.2. Total Phenolic Content (TPC)

The Total Phenolic Content (TPC) of root and leaflet extracts from *E*. *oleracea* ranged from 14 to 84 µg Gallic acid equivalent/mg Dry Matter (µg GAEq/mg DM) (Table 1). 

Best results were found with acetone independently of the organ used. The highest values were found for the leaflet extracts of *E*. *oleracea*, with 84 µg GAEq/mg DM, which was two-fold higher than the TPC of palm berries including *E*. *oleracea*, *O*. *bataua* [14] and *O*. *bacaba* [15], known to be antioxidants. The TPC of *E*. *oleracea* roots were globally similar to that of *Oenocarpus* roots extracts [12], whereas *E*. *oleracea* leaflets were richer in polyphenols than *Oenocarpus* leaflets [12]. Moreover, the TPC of *E*. *oleracea* leaflets was almost similar to the TPC of green tea leaves (100–125 µg GAEq/mg), which has one of the strongest recorded antioxidant activities. There was a high correlation between the total polyphenolic content (TPC) and the antioxidant activity in the different assays (*p* < 0.05) with high Pearson’s r correlation coefficients between TPC and DPPH (0.945), TPC and FRAP (0.983), TPC and ORAC (0.986), and TPC and CAA activity (0.968) (Figure S1).

### 2.3. Identification of Compounds in Root and Leaflet Extracts by LC-MS/MS

**Figure 2:**
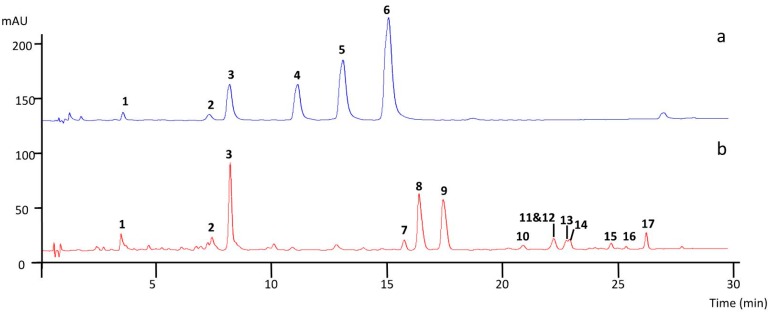
Representative chromatograms of: (**a**) root extracts; and (**b**) leaflet extracts of *Euterpe oleracea* at λ = 320 nm; Kinetex PFP column 100 × 4.6 mm, 2.6 µm; (**1**) 3-CQA; (**2**) 4-CQA; (**3**) 5-CQA; (**4**) 4-CSA; (**5**) 5-CSA; (**6**) CSA; (**7**) 6,8-di-*C*-hexosyl apigenin; (**8**,**9**) 6,8-di-*C*-hexosyl apigenin sulfate; (**10**,**14**,**15**) 6-*C*-hexosyl-8-*C*-pentosyl apigenin isomers; (**12**,**16**) 6-*C*-pentosyl-8-*C*-hexosyl apigenin isomer; (**11**) 8-*C*-glucosyl luteolin; (**13**) 6-*C*-glucosyl luteolin; and (**17**) 6-*C*-glucosyl apigenin; CQA: caffeoylquinic acid; CSA: caffeoylshikimic acid.

**Figure 3:**
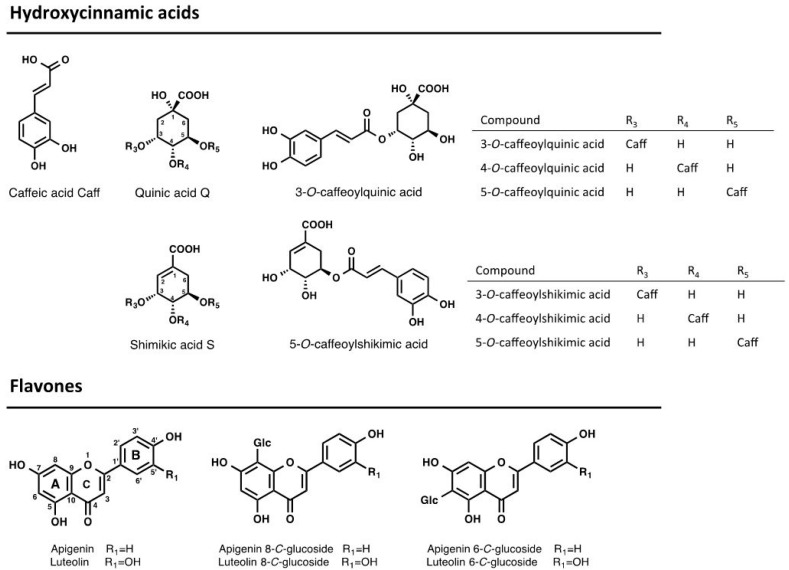
Structure of main components of root and leaflet extracts of *Euterpe oleracea*; Glc: glucose.

The UV chromatograms recorded at 320 nm showed that both *E*. *oleracea* root and leaflet extracts (Figure 2) contained polyphenols, whose structures (Figure 3) were then determined by LC-MS/MS. Leaflet extracts showed more complex polyphenol profiles (fourteen compounds) than root extracts (six compounds), while some peaks (**1**, **2**, and **3**) were common to both extracts.

#### 2.3.1. Characterization of Caffeoylquinic Derivatives (*M*r = 354) in Root and Leaflet Extracts

In the root and leaflet extracts, three peaks (**1**, **2**, and **3**) at retention time (*t*_R_) 4.2, 7.2 and 9.1 min yielded [M − H]^−^ ions at *m*/*z* 353 and [M + Na]^+^ at *m*/*z* 377 and showed UV spectra (at λ = 298 nm and 320 nm) characteristic of caffeoylquinic acids (CQA, *M*r = 354).

In positive mode, the ions at *m*/*z* 163 and 145, produced by the loss of the quinic acid moiety [19] were not conclusive of the structure of the CQA. The structure of CQA isomers was assigned according to their diagnostic ions at *m*/*z* 191, 179 and 173 in the negative mode [20]. Compound **2** (*t*_R_ 7.2 min) was assigned to 4-CQA, having a base peak at *m*/*z* 173 in the MS^2^ spectrum. Compounds **1** (*t*_R_ 4.2 min) and **3** (*t*_R_ 9.1 min), both having *m*/*z* 191 as a base peak were distinguished by the intense ion at *m*/*z* 179 in the MS^2^ spectrum, characteristic of 3-CQA for compound **1**, whereas compound **3** was assigned to 5-CQA (Table 2). The retention order of the CQA isomers (**3** < **4** < **5**) was similar to the study of Regos and Treutter, 2010 [21] using a similar PFP column.

#### 2.3.2. Characterization of Caffeoylshikimic Derivatives (*M*r = 336) in Root Extracts

In the root extracts, three peaks (**4**, **5**, **6**) at *t*_R_ 12.2, 14.2 and 16.2 min yielded [M − H]^−^ ions at *m*/*z* 335, [M + Na]^+^ at *m*/*z* 359, and showed UV spectra (λ = 298 nm and 320 nm) corresponding to caffeoylshikimic acids (CSA, *M*r = 336). In positive mode, the ions at *m*/*z* 163 and 145 were characteristic of the caffeoyl moiety [19]. The structure of CSA was tentatively assigned according to their diagnostic ions at *m*/*z* 317, 291, 179, 161 and 135 in negative mode [22,23,24] (Table 2). The fragmentation pattern of compound **4** with an intense ion at *m/z* 161 was conclusive of a 4-CSA. The absence of the ion at *m*/*z* 161 in the MS^2^ spectrum of compound **5** and the presence of ions at *m*/*z* 317 and *m*/*z* 291 was indicative of a 5-CSA isomer. The fragmentation pattern of compound **6** was close to that of a 4-CSA isomer but was not conclusive of the structure.

#### 2.3.3. Characterization of Apigenin Derivatives (*M*r = 432, Mr = 594, *M*r = 674, and *M*r = 564) in Leaflet Extracts

The UV spectra of eleven compounds (**7**–**17**) in the leaflet extracts of *E*. *oleracea* showing a λ_max_ at 340 nm were indicative of flavone derivatives. Compound **17** (*M*r = 432) gave [M−H]^−^ at *m*/*z* 431 and [M + H]^+^ at *m*/*z* 433. Characteristic losses of 90, 120 and 150 Da were diagnostic of hexose residues. The fragment ions at *m*/*z* 311 (aglycone + 42) and *m*/*z* 341 (aglycone + 72) in negative mode clearly indicate 6-*C*-glycosyl apigenin [25], whose identity was confirmed with a synthetic standard. Nine compounds, i.e., compounds **7** (*M*r = 594), **8**, **9** (*M*r = 674) **10**, **12**, **14**, **15**, and **16** (*M*r = 564), showed the characteristic MS^2^ fragmentation of di-*C*-glycosyl flavones, the occurrence of fragment ions at *m*/*z* 353 (aglycone + 83) and 383 (aglycone + 113) being indicative of apigenin derivatives [25]. Compound **7** (Mr = 594) yielded [M − H]^−^ at *m*/*z* 593, with a fragmentation pattern in negative mode characteristic of hexose derivatives (losses of 90 Da and 120 Da) and was assigned to a 6,8-di-*C*-hexosyl apigenin. Compounds **8** and **9** (*M*r = 674) yielded [M − H]^−^ at *m*/*z* 673 and [M + K]^+^ at 713. The MS^2^ spectrum showed a first loss of 80 Da, characteristic of sulfates, followed by successive losses of 90 Da, 120 Da and 150 Da, characteristics of hexose residues in both positive and negative modes. Compounds **8** and **9** were tentatively assigned to 6,8-di-*C*-hexosyl apigenin sulfates. The lack of a hypsochromic shift in the band I of **8** and **9** compared to compound **7** suggest the sulfation occurred on position **7** or on the sugar moieties [26]. Six peaks (**10**, **12**, **14**, **15**, **16**) (*M*r = 564) at *t*_R_ 21.2, 22.3, 23.2, 24.9 and 25.6 min yielded [M − H]^−^ ions at *m*/*z* 563 in the negative mode. Their MS^2^ spectrum showed losses of 60 Da, 90 Da, 120 Da and 150 Da, characteristic of pentose and hexose residues. They were assigned as 6,8-di-*C*-pentosyl-hexosyl apigenin derivatives. The [M − H-60]^−^ ion resulting from the fragmentation of the pentose moiety was more intense in **12** (100%) and **16** (25%) than in other compounds, indicating that the pentose moiety was on position 6, which fragments preferentially over sugars on position 8 [25]. Therefore, compounds **12** and **16** were identified as 6-*C*-pentosyl-8-*C*-hexosyl apigenin isomers. The others compounds (**10**, **14**, and **15**) were assigned as 6-*C*-hexosyl-8-*C*-pentosyl apigenin isomers.

#### 2.3.4. Characterization of Luteolin Derivatives (*M*r = 448)

Compounds **11** and **13** (*M*r = 432) gave [M − H]^−^ at *m*/*z* 447 and [M + H]^+^ at *m*/*z* 449. Characteristics losses of 90 Da and 120 Da were diagnostics of hexose residues. The fragments ions at *m*/*z* 327 (aglycone + 42) and *m*/*z* 357 (aglycone + 72) in the negative mode clearly indicated *C*-glycosyl luteolin derivatives [25]. Based on retention order and injection of synthetic standards, **11** was identified as 8-*C*-glycosyl luteolin and **13** as 6-*C*-glycosyl luteolin.

#### 2.3.5. Quantification of Chemical Compounds in Root and Leaflet Extracts

**Figure 4:**
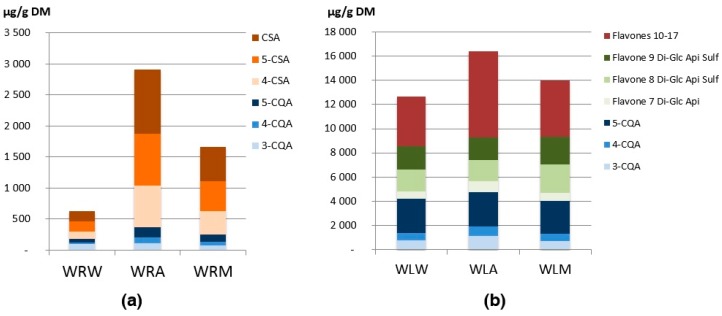
Chemical composition of: (**a**) root extracts; and (**b**) leaflet extracts of *Euterpe oleracea*; W: Wassaye (*E*. *oleracea*); R: roots; L: leaflets; W: water; A: acetone/water 70/30; M: methanol/water 70/30; CQA: caffeoylquinic acid; CSA: caffeoylshikimic acid (for identification of compounds **7**–**17**, see Table 2); Di-Glc-Api: 6,8-di-*C*-hexosyl apigenin; Di-Glc-Api Sulf: 6,8-di-*C*-hexosyl apigenin sulfate; *n* = 3 biological replicates.

Root extracts of *E*. *oleracea* were mainly characterized by the presence of hydroxycinnamic acids HCA, namely three caffeoylquinic acids (CQA) and three caffeoylshikimic acids (CSA) (Figure 4). Hydroxycinnamic acids are known to display biological properties such as antiviral, anti-inflammatory and antioxidant activity [27]. They also play important physiological role of defense against pathogens and disease resistance [27], which is essential for vulnerable vegetative organs such as roots. The CSA content was higher than the CQA content in the root extracts, (2.5 mg/g DM against 0.5 mg/g DM for the most concentrated extract), i.e., 70%–85% of all HCA (Figure 4). 5-CQA was the major compound amongst CQA, while each of the three CSA was found in almost similar amounts. The qualitative composition of *E*. *oleracea* roots was similar to that of *O*. *bataua* and *O*. *bacaba* [12]. However, the quantitative compositions of *O*. *bacaba* were different with a global two-fold lower content in compound compared to *E*. *oleracea*.

Leaflet extracts were characterized by two main compound families: three caffeoylquinic acids, as in the root extracts, and eleven flavones (Figure 4). 5-CQA was the main CQA in the leaflet extracts (40%–70% of total CQA). Higher contents of CQA (up to 4.5 mg/g DM) were found in the leaflet extracts compared to the root extracts. Leaflet flavones were quantified mainly as *C*-monoglycosyl and *C*-diglycosyl derivatives of apigenin, and to a lesser extent as *C*-glycosyl derivatives of luteolin. Di-*C*-glycosyl derivatives of apigenin (compounds **7**–**9**) were the major compounds in leaflet extracts of *E*. *oleracea*, accounting for almost 50% of all *C*-glycosylflavones, the sulfated flavones **8** and **9** being the major ones with about 2 mg/g DM. Apigenin *C*-glycosides are phytochemical markers specific to a few *Arecaceae* species, while *C*-glycosyl luteolin derivatives are more common amongst palm tree species [28]. More recently, *C*-glycosyl derivatives of apigenin, close to those found in this work, were identified in another Amazonian palm tree species, *Mauritia flexuosa* [29]. The presence of sulfated flavones in the leaflet extracts of *E*. *oleracea* is interesting as natural sulfated flavonoids inspire the design of smaller bioactive compounds with sulfate groups [30]. *E*. *oleracea* leaflets composition was qualitatively similar to that of *Oenocarpus* leaflets [12] but the quantitative composition was conspicuously different (maximum 1500 and 5000 µg/g DM for *O*. *bataua* and *O*. *bacaba*, respectively, against 16000 µg/g DM for *E*. *oleracea*). It was interesting to observe that these three palm tree species (*E*. *oleracea*, *O*. *bacaba* and *O*. *bataua*) have the same qualitative composition of their roots or of their leaflets but have quite different antioxidant activities. Indeed, *E*. *oleracea* leaflets were two fold more active than *O*. *bacaba* and *O*. *bataua* leaflets. The difference of activity could come from the quantity of polyphenolic compounds in the extracts, rather than the qualitative composition. These results interrogate the fact that there might be a particular regulation of key enzymes in the biosynthetic pathway of polyphenols in *E*. *oleracea* leaflets. To validate this hypothesis, numerous molecular and biochemical studies will be required.

### 2.4. Relationship between the Chemical Composition and the Antioxidant Activity

**Figure 5:**
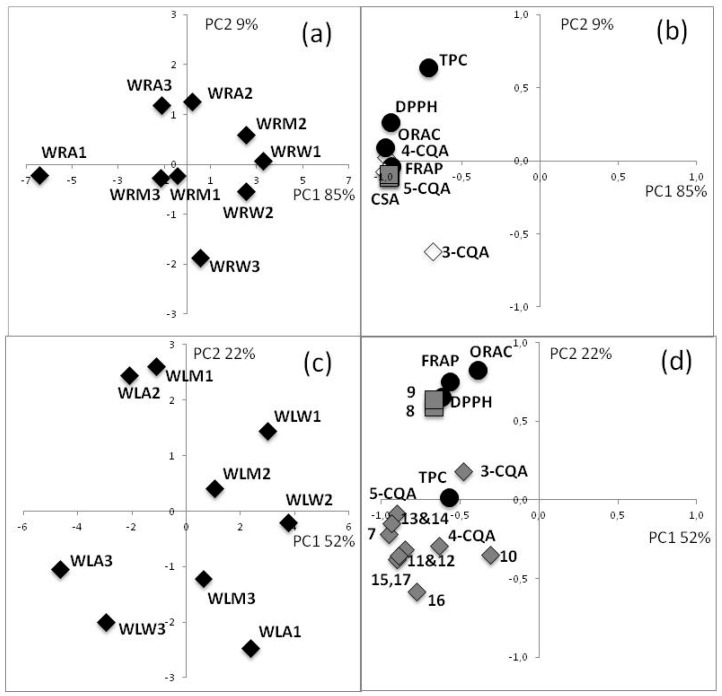
Principal Component Analysis (PCA) plots for antioxidant activity and chemical composition of: root extracts (**a**,**b**); and leaflet extracts (**c**,**d**) of *Euterpe oleracea*; W: Wassaye (*E*. *oleracea*); R: roots; L: leaflets; W: water; A: acetone/water: 70/30; M: methanol/water: 70/30; CQA: caffeoylquinic acid; CSA: caffeoylshikimic acid, (for identification of compounds **7**–**17**, see Table 2); DPPH: 2,2-Diphenyl-1-picrylhydrazyl; FRAP: ferric reducing antioxidant power; ORAC: oxygen radical absorbance capacity.

Principal Component Analysis (PCA) was carried out using the data from the chemical antioxidant assays (DPPH, FRAP, ORAC) and the quantification of compounds in root and leaflet extracts of *E*. *oleracea*. The antioxidant activity of the roots was linked to the CSA and CQA contents (negative side of PC1) (Figure 5). There was no clear differentiation of the methanol and acetone root extracts according to the PCA, as they had very similar antioxidant activity and chemical composition. 

For the leaflet extracts, the most active extracts in all the chemical assays were on the positive side of PC2, which was correlated with high contents of flavones **8** and **9** (Figure 5). These results translated quite well to the CAA assay, where the leaflet extracts of *E*. *oleracea* were very active. The PCA highlighted a correlation between the antioxidant activity of *E*. *oleracea* leaflet extracts and the presence of di-*C*-glycosyl apigenin sulfates (**8** and **9**). Indeed, hydroxyl substituted flavones such as apigenin or luteolin derivatives are known to have high antioxidant activity due to their structures [31].

## 3. Materials and Methods

### 3.1. Chemicals and Reagents

Solvents used for extraction and LC-MS analysis were of HPLC grade, obtained from Carlo Erba Reagents (Val de Reuil, France). Standards of 5-*O*-caffeoylquinic acid, orientin (>99%), isoorientin (>99%), and isovitexin (>99%) were obtained from Extrasynthèse (Genay, France). Gallic acid-1-hydrate (99%) was purchased from Panreac Quimica (Barcelona, Spain).

### 3.2. Plant Materials

Roots and leaflets of three specimens of *E*. *oleracea* were harvested on February 2013, in Macouria, French Guiana. Samples were washed, cut, freeze-dried and grinded immediately after collection. Dried matter was then stored at −20 °C to limit degradation until extraction.

### 3.3. Extraction

Extractions were performed following the method previously published [12]. Three solvents were used for extraction of roots and leaflets from *E*. *oleracea* and green tea leaves: water (W), methanol/water 70/30 *v*/*v* (M) and acetone/water 70/30 *v*/*v* (A). The combination of acetone or methanol with water is commonly used to extract polyphenols [32]. Ultrasounds were used to assist the extraction of polyphenols of leaflets and roots from *E*. *oleracea*, as they are known to be a green alternative to conventional extraction methods and give optimized extraction efficiency and reduced extraction time [33]. Preliminary studies using ultrasound extraction were carried out to determine that four successive extractions gave optimized extraction efficiency.

### 3.4. Total Phenolics by Folin–Ciocalteu

Total Phenolic Content (TPC) was determined using the Folin–Ciocalteu method as previously described [12].

### 3.5. Chemical Antioxidant Assays (DPPH, FRAP, ORAC)

Chemical antioxidant properties were assessed using DPPH, FRAP and ORAC assays as described in our previous paper [12]. These assays are based on different antioxidant mode of action like electron transfer (FRAP), hydrogen transfer (ORAC) or a mixed mechanism implying both electron and hydrogen transfer (DPPH) [13].

### 3.6. Cellular Assay

#### 3.6.1. Cell Culture

Normal Human Dermal Fibroblasts (NHDF) were purchased from PromoCell (Heidelberg, Germany), cultured in growth medium RPMI GlutaMAX™ (89%) (Thermo Fischer Scientific, Waltham, MA, USA), supplemented with 5% FBS (Fetal Bovine Serum), 1% antibiotics, 5% CO_2_ and maintained at 37 °C. NHDF cells were used to determine the cytotoxicity of *E*. *oleracea* extracts and to assess the antioxidant activity of the extracts in a cell-based assay described in Section 3.6.3.

#### 3.6.2. Cytotoxicity Assay

Cytotoxicity of the extracts in NHDF was measured using the MTT (3-(4,5-dimethylthiazol-2-yl)-2,5-diphenyltetrazolium bromide) method [34]. NHDF cells were incubated for 24 h at 37 °C, in presence or absence of *E*. *oleracea* extracts at 100, 200, 300, 400 and 500 µg/mL. The cells were washed twice with 500 µL of a phosphate buffer solution (PBS), then incubated at 37 °C for 3 h with MTT at 0.5 mg/mL. MTT was removed using 1 mL of DMSO and the plate was kept in the dark at room temperature during 30 min before recording the absorbance at 595 nm using a plate-reader (Dynex, Magellan Biosciences, Tampa, FL, USA) to detect viable cells. A decrease of the absorbance by more than 20% was set as the limit for cytotoxicity.

#### 3.6.3. Cellular Antioxidant Activity (CAA) Assay

The cell-based assay was done according to the method of Wolfe and Liu [35], with some modifications. Briefly, NHDF cells were seeded for 24 h on a 96-well microplate at a density of 11 × 10^4^ cells by well in 100 µL of RPMI growth medium. The growth medium was removed and the wells were washed with PBS. Wells were treated in triplicates with 100 µL of extracts at four concentrations (10, 25, 50 and 100 µg/mL), not cytotoxic to the NHDF cells, plus 50 µM 2′,7′-dichlorodihydrofluorescein diacetate (DCFH-DA) dissolved in the treatment medium and kept in the dark for 1 h. Control wells (cells treated with 50 µM of DCFH-DA, without extract), and blank wells (cells treated only with growth medium) were included. Then, the wells were drained from the treatment medium and 100 µL of 2,2′-Azobis(2-amidinopropane) dihydrochloride (AAPH) at 250 µM was added in each well (except in blank wells). Immediately after the AAPH addition, the plate was placed into a plate-reader (Dynex, Magellan Biosciences) at 37 °C and the fluorescence was recorded for 30 min (λ_excitation_ 485 nm, λ_emission_ 538 nm). After blank subtraction from the fluorescence readings, the area under the curve of fluorescence versus time was integrated to calculate the CAA value at each extracts concentration tested as follows: CAA unit: 100 − (∫ SA/∫ CA) × 100 where ∫ SA is the integrated area under the sample fluorescence versus time curve and ∫ CA is the integrated area from the control curve. The median effective concentration EC_50_ (µg/mL), was determined for *E*. *oleracea* extracts from the median effect plot of log (ƒa/ƒu) versus log (concentration), where ƒa is the fraction affected and ƒu is the fraction unaffected by the treatment. Quercetin was used as a standard in the CAA assay and the cellular antioxidant activity of *E*. *oleracea* extracts was then expressed in µmol Quercetin equivalents/g of dried matter (µmol QEq/g DM). 

#### 3.6.4. DNA Nicking Assay

The DNA nicking assay was performed using the pUC18 plasmid. All conditions required to analyze aqueous and organic extracts were previously optimized [16]. Four microliters of *E*. *oleracea* extracts (max 7% acetone) at various concentrations were added to 4 µL of the Fenton reaction mixture, containing: plasmid DNA (150 µg/µL), phosphate buffer (50 mM, pH 7.4), H_2_O_2_ (30 mM), FeSO_4_ (2 mM/8 mM) and EDTA-Na_2_ (3.75 mM/15 mM) for aqueous and acetone extracts respectively. The final volume was adjusted to 24 µL using distilled water and incubated for 20 min and 15 min at 37 °C for aqueous and acetone extracts respectively. Two microliters of loading dye was added to the incubated mixture, and 10 µL were loaded onto 1% (*w*/*v*) agarose gel. After electrophoresis in TAE buffer (0.04 M tris-acetate and 1 mM EDTA, pH 7.4) using a DNA subcell (Bio-Rad Laboratories, Hercules, CA, USA), the agarose gel was stained with ethidium bromide for 15 min and DNA bands were analyzed using a Bio-Rad Gel Doc™ XR (Bio-Rad Laboratories). The prooxidant and antioxidant activity of the extracts was assessed using band intensity (NIH Image J) of form I (supercoiled DNA) and form III (nicked linear DNA) compared to the appropriate controls. DNA incubated with and without Fenton’s reagent was used as positive and negative control, respectively. Trolox was used as a control of DNA protection, at a concentration of 0.01 and 0.1 mg/mL for aqueous and acetone extracts, respectively.

#### 3.6.5. Analysis by LC-MS/MS

LC-MS/MS analysis was performed on an ion trap (500-MS Varian, Palo Alto, CA, USA) equipped with an electrospray ionization source and coupled to a Pro Star LC system (Agilent Technologies, Santa Clara, CA, USA) and a Pro Star 335 PDA (Agilent). Extracts were filtered using 0.2 µm PTFE filters. Twenty microliters of samples were injected onto a Kinetex PFP column, 100 × 4.6 mm, 2.6 µm (Agilent) using a gradient of 1% formic acid (A) and acetonitrile (B) at 25 °C. The gradient was as follows: 5%–10% B in 10 min, 10%–20% in 10 min, 20% held for 10 min, 20%–100% in 5 min, 100% held for 5 min, and returned to initial conditions for 9 min to reequilibrate the column. The flow rate was 1 mL/min. The total run time per sample was 60 min.

Analyses were first performed using Full scan mode (*m*/*z* 120–750), both in negative mode and positive mode to identify the molecular ions and then in TurboDDS^™^ mode (Data Dependent Scanning, Varian) to acquire MS^2^ spectra. Helium was used as the damping and collision gas at 0.8 mL/min. The operation parameters were as follows: nebulizer gas pressure 50 psi, drying gas pressure 25 psi, drying gas temperature 350 °C, needle voltage −5000 V/5000 V, sprayshield voltage −600 V, spray chamber 50 °C, capillary voltage 100 V/−70 V (negative ionization mode/positive ionization mode).

The identification of compounds was performed according to their MS^2^ fragmentation [15,16,17,26,27], using standards when available. Quantification was carried out with UV detection. 5-CQA and 6-*C*-glycosyl apigenin (isovitexin) were used as calibration standards (λ_max_ = 320 nm) to quantify hydroxycinnamic acids (caffeoylquinic acids—CQA and caffeoylshikimic acids—CSA) and flavones, respectively. Gallic acid was used as the internal standard at λ_max_ = 280 nm.

#### 3.6.6. Statistical Data Analysis

Results of antioxidant activity were presented as the mean of three biological replicates and standard error (SE) or standard error of the mean (SEM). Comparisons between extracts in the TPC, ORAC, FRAP, DPPH and DNA nicking assays were performed using an ANOVA followed by multiple comparisons using Fisher’s Least Significant Difference test (*p* < 0.05). Principal component analysis (PCA) was performed on the phytochemical composition and chemical antioxidant activity (DPPH, FRAP, and ORAC) of roots and leaflet extracts from *E*. *oleracea* using the Statistica program (Statsoft, Paris, France).

## 4. Conclusions

Root and leaflet extracts from *E*. *oleracea* were active across the board in different chemical antioxidant assays (DPPH, FRAP, ORAC, DNA nicking) and biological assay (CAA). The high antioxidant activity of the root and leaflet extracts of *E*. *oleracea* was respectively correlated to the presence of hydroxycinnamic acids and apigenin *C*-glycosides. The high antioxidant activity of *E*. *oleracea* leaflets seemed to come from their highest content of polyphenols, suggesting a particular regulation of the polyphenols biosynthetic pathway. This study shows for the first time that *Euterpe oleracea* roots and leaflets, which are currently by-products of the palm heart industry, could, along with the berries, be valorized as a new non-cytotoxic source of antioxidants containing hydroxycinnamic acids and flavonoids for pharmaceutical, nutraceutical or cosmetic applications.

## Figures and Tables

**Table 1:** Antioxidant activity and total phenolic content (TPC) of root and leaflet extracts of *Euterpe oleracea*.

Extract Name (Plant/Part/Solvent)	TPC (µg GAEq/mg DM) *	DPPH (µmol TEq/g DM) *	FRAP (µmol Fe(II) Eq/g DM) *	ORAC (µmol TEq/g DM) *	EC_50_ in NHDF (µg/mL)	CAA in NHDF (µmole QEq/g DM)
WRW	14 ± 2 ^d^	105 ± 5 ^c^	310 ± 129 ^e^	302 ± 124 ^d^	30 ± 7	10 ± 2
WRA	58 ± 9 ^b,c^	471 ± 101 ^b^	769 ± 137 ^c,d^	1259 ± 320 ^c^	14 ± 2	29 ± 4
WRM	28 ± 4 ^c,d^	237 ± 72 ^c^	449 ± 111 ^d,e^	686 ± 186 ^d^	16 ± 1	14 ± 1
WLW	62 ± 7 ^a,b^	479 ± 39 ^b,c^	965 ± 178 ^b,c^	1643 ± 385 ^b,c^	29 ± 9	45 ± 12
WLA	84 ± 7 ^a^	991 ± 81 ^a^	1381 ± 376 ^a^	2229 ± 484 ^a^	10 ± 1	208 ± 23
WLM	76 ± 7 ^a,b^	660 ± 72 ^b^	1194 ± 180 ^a,b^	2177 ± 611 ^a,b^	16 ± 2	87 ± 12
TLW ^e^	100 ± 9	748 ± 52	1911 ± 82	1348 ± 45	-	-
TLA ^e^	126 ± 2	1185 ± 34	2686 ± 107	2167 ± 19	-	-
TLM ^e^	98 ± 8	1045 ± 69	2677 ± 142	2366 ± 31	-	-
ObtLA ^f^	50	460	730	1200	14	100
ObcLA ^f^	60	540	1030	1560	24	50
ObcBA ^g^	30	240	200	170	-	-
WBA ^g^	40	240	130	430	-	-

* Averages with the same letter ^a–d^ within columns are not significantly different (*p* < 0.05) using Fisher’s Least Significant Difference test; W: Wassaye (*E*. *oleracea*); Obt: *O*. *bataua*; Obc: *O*. *bacaba*; R: roots; L: leaflets; B: berries; T: Green Tea leaves; W: water; A: acetone/water 70/30; M: methanol/water 70/30; CAA: cellular antioxidant activity; NHDF: normal human dermal fibroblasts; GAEq: Gallic acid equivalent; TEq: Trolox equivalent; QEq: Quercetin equivalent; DM: dry matter; TPC: total phenolic content; EC_50_: median effective concentration; CAA: cellular antioxidant activity; DPPH: 2,2-Diphenyl-1-picrylhydrazyl; FRAP: ferric reducing antioxidant power; ORAC: oxygen radical absorbance capacity; ^e^ data from Leba et al., 2014 [16]; ^f^ data from Leba et al., 2016 [12]; ^g^ data from Rezaire et al., [14].

**Table 2:** Identification of main components of root and leaflet extracts of *Euterpe oleracea*.

*N*	*t*_R_ (min)	UV λ_max_ (nm)	Negative Mode MS	Negative Mode MS^2^ (%)	Positive Mode MS	Positive Mode MS^2^ (%)	Tentative Identity	Abbreviation	Extracts
1	4.2	238, 295sh, 321	353 [M − H]^−^	MS^2^[353]: 191 (100), 179 (25)	377 [M + Na]^+^	377/353/163/145	3-Caffeoylquinic acid	3-CQA	Roots, leaflets
2	7.9	237, 286sh, 322	353 [M − H]^−^	MS^2^[353]: 173 (100)	377 [M + Na]^+^	377/353/163/145	4-Caffeoylquinic acid	4-CQA	Roots, leaflets
3	9.1	239, 295sh, 323	353 [M − H]^−^	MS^2^[353]: 191 (100)	377 [M + Na]^+^	377/353/163/145	5-Caffeoylquinic acid	5-CQA	Roots, leaflets
4	12.2	239, 295sh, 325	335 [M − H]^−^	MS^2^[335]: 291 (100), 179 (60), 161 (80), 135 (75)	359 [M + Na]^+^	359/163/145	4-Caffeoylshikimic acid	4-CSA	Roots
5	14.2	239, 295sh, 325	335 [M − H]^−^	MS^2^[335]: 317 (100), 291 (100), 179 (50)	359 [M + Na]^+^	359/163/145	5-Caffeoylshikimic acid	5-CSA	Roots
6	16.2	239, 295sh, 325	335 [M − H]^−^	MS^2^[335]: 317 (5), 291 (5), 179 (75), 161 (100), 135 (80)	359 [M + Na]^+^	359/163/145	Caffeoylshikimic acid	CSA	Roots
7	16.1	239, 270, 335	593 [M − H]^−^	MS^2^[593]: 503 (20), 473 (25), 383 (50), 353 (50)	595 [M + Na]^+^	595/577/457/427/317	6,8-di-*C*-hexosyl apigenin	Di-Glc-Api	Leaflets
8	16.8	239, 270, 335	673 [M − H]^−^	MS^2^[673]: 593 (100), 575 (10), 503 (10), 473 (15), 413 (5), 383 (10), 353 (10) MS^2^[593]: 503 (25), 473 (10), 383(50), 353 (100) MS^3^[673→593]: 473(100), 413(20), 383(60), 353(100) MS^3^[673→473]: 383 (100), 353 (100)	713 [M + Na]^+^	MS^2^[713]: 633 (100), 593 (25), 543 (25), 513 (25), 423 (10), 393 (10), 351 (10) MS^3^[713→633]: 573 (100), 543 (100), 513 (100), 483 (20), 423 (75), 393 (20), 351 (50)	6,8-di-*C*-hexosyl apigenin sulfate	Di-Glc-Api Sulf	Leaflets
9	17.8	239,270,335	673 [M − H]^−^	MS^2^[673]: 593 (100), 503 (10), 473 (20), 383 (10), 353 (10) MS^2^[593]: 473 (60), 383 (30), 353 (100) MS^3^[673→593]: 473 (100), 383 (50), 353 (100) MS^3^[673→473]: 383 (100), 353 (100)	713 [M + K]^+^	MS^2^[713]: 633 (100), 593 (25), 543 (25), 513 (75), 483 (25), 423 (30), 393 (30), 363 (10), 351 (10) MS^3^[713→633]: 573 (20), 543 (100), 513 (70), 483 (5), 423 (50), 393 (20), 381 (50), 351 (25)	6,8-di-*C*-hexosyl apigenin sulfate	Di-Glc-Api Sulf	Leaflets
10	21.2	238,272,339	563 [M − H]^−^	MS^2^[563]: 473 (10), 443 (30), 383 (80), 353 (80)	-	-	6-*C*-hexosyl-8-*C*-pentosyl apigenin isomer		Leaflets
11	22.1	240,270,342	447 [M − H]^−^	447/429/411/357/327/299	449 [M + H]^+^	449/413/383	8-*C*-glycosyl luteolin (orientin) *		Leaflets
12	22.3	238,272,339	563 [M − H]^−^	MS^2^[563]: 503 (100), 473 (10), 443 (20), 413 (75), 383 (50), 353 (10)	-	-	6-*C*-pentosyl-8-*C*-hexosyl apigenin isomer		Leaflets
13	22.9	241,270,346	447 [M − H]^−^	447/429/411/357/327/299	449 [M + H]^+^	MS^2^[449]: 431 (10), 413 (80), 395 (20), 383 (50), 353 (60), 329 (10), 299 (100)	6-*C*-glycosyl luteolin (isoorientin) *		Leaflets
14	23.2	238,272,340	563 [M − H]^−^	MS^2^[563]: 473 (75), 443 (75), 383 (25), 353 (100)	-	-	6-*C*-hexosyl-8-*C*-pentosyl apigenin isomer		Leaflets
15	24.9	241,271,336	563 [M − H]^−^	MS^2^[563]: 443 (100), 383 (30), 353 (50), 323 (20)	-	-	6-*C*-hexosyl-8-*C*-pentosyl apigenin isomer		Leaflets
16	25.6	238,277,335	563 [M − H]^−^	MS^2^[563]: 503 (25), 473 (10), 383 (10), 353 (100)	-	-	6-*C*-pentosyl-8-*C*-hexosyl apigenin isomer		Leaflets
17	26.4	241,270,337	431 [M − H]^−^	431/413/395/341/311/283	433 [M + H]^+^	MS^2^[433]: 397 (75), 379 (30), 367 (100), 337 (50), 313 (40), 295 (20), 283 (50)	6-*C*-glycosyl apigenin (isovitexin) *		Leaflets

* Structure confirmed using standard compounds.

## Supplementary Materials

Supplementary materials can be found at www.mdpi.com/1422-0067/18/1/61/s1.

## Abbreviations

DPPH	2,2-Diphenyl-1-picrylhydrazyl
FRAP	Ferric Reducing Antioxidant Power
ORAC	Oxygen Radical Absorbance Capacity
CAA	Cellular Antioxidant Activity
TPC	Total Polyphenol Content
GAEq	Gallic acid equivalent
TEq	Trolox equivalent
QEq	Quercetin equivalent
DM	Dry Matter
PCA	Principal Component Analysis
NHDF	Normal Human Dermal Fibroblasts
HCA	Hydroxycinnamic acid
CQA	Caffeoylquinic acid
CSA	Caffeoylshikimic acid
MTT	3-(4,5-Dimethylthiazol-2-yl)-2,5-diphenyltetrazolium bromide
