# Supplementary material for: Chemical Composition and Antioxidant Activity of Euterpe oleracea Roots and Leaflets
Source: Int J Mol Sci. 2016 Dec 29;18(1):61. doi: 10.3390/ijms18010061 (PMC5297696; doi:10.3390/ijms18010061)
Supplement: Supplementary file 1 [file ijms-18-00061-s001.pdf]

Supplementary Materials: Chemical Composition and Antioxidant Activity of *Euterpe oleracea* Roots and Leaflets

Christel Brunschwig, Louis-Jérôme Leba, Mona Saout, Karine Martial, Didier Bereau and Jean-Charles Robinson

Table S1. Cytotoxicity of *Euterpe oleracea* extracts in NHDF cells

Extract Name (Plant/Part/Solvent)	Cytotoxicity NHDF (μg/mL) [a]
WRW	>500
WRA	200
WRM	300
WLW	500
WLA	>500
WLM	>500
ObtLA [b]	500
ObcLA [b]	200

W: Wassaye (*E. oleracea*); Obt: O. bataua; Obc: O. bacaba; R: roots; L: leaflets; B: berries; T: Green Tea leaves; W: water; A: acetone/water: 70/30; M: methanol/water: 70/30; CAA: cellular antioxidant activity; NHDF: normal human dermal fibroblasts; [a] concentration at which less than 80% viable cells were observed (concentrations tested: 100, 200, 300, 400, 500 μg/mL); [b] data from Leba et al., 2016 [12].

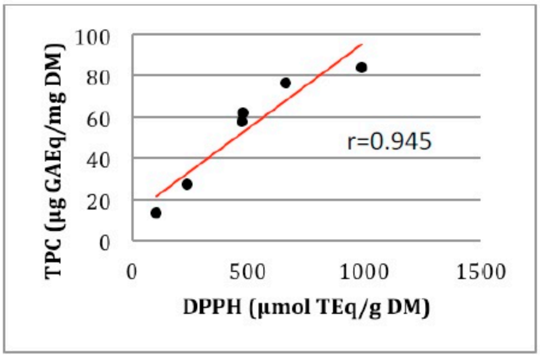

(a)

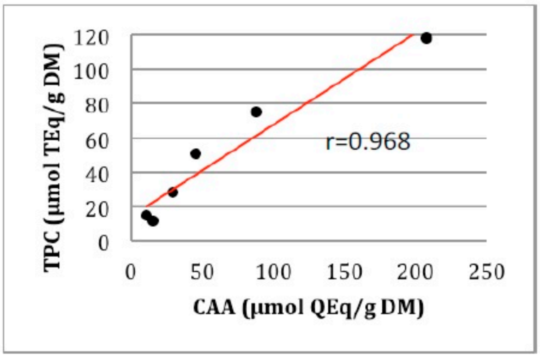

(d)

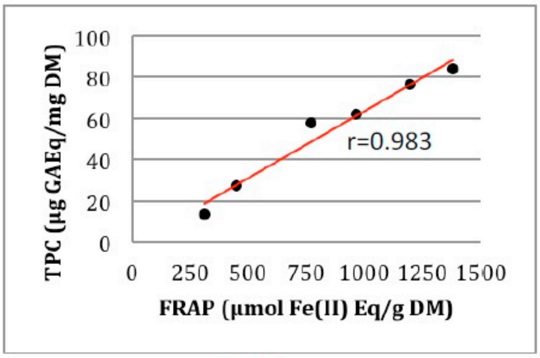

(b)

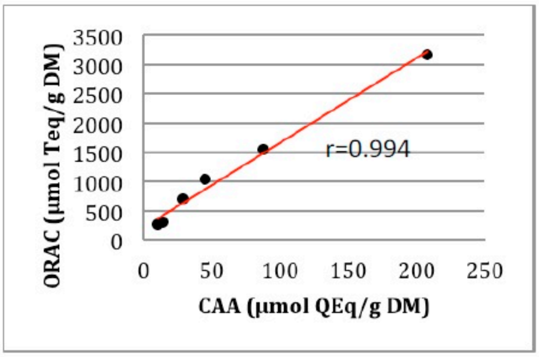

(e)

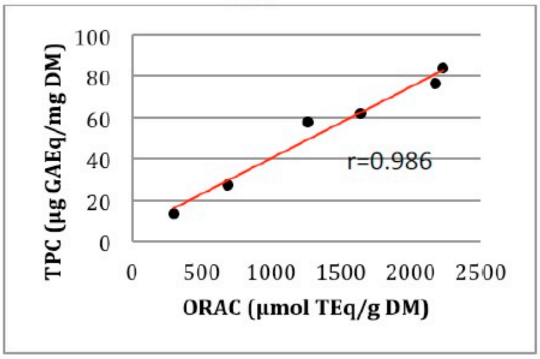

(c)

Figure S1. Correlations ($p < 0.05$) indicating Pearson's r correlation coefficients between (**a**) TPC and DPPH; (**b**) TPC and FRAP; (**c**) TPC and ORAC; (**d**) TPC and CAA; and (**e**) ORAC and CAA; TPC: total phenolic content; DPPH: 2,2-Diphenyl-1-picrylhydrazyl; FRAP: ferric reducing antioxidant power; ORAC: oxygen radical absorbance capacity; CAA: cellular antioxidant activity; TEq: Trolox equivalent; QEq: Quercetin equivalent.
